# The Relationship Between Auditory-Motor Integration, Interoceptive Awareness, and Self-Reported Stuttering Severity

**DOI:** 10.3389/fnint.2022.869571

**Published:** 2022-05-06

**Authors:** M. Florencia Assaneo, Pablo Ripollés, Seth E. Tichenor, J. Scott Yaruss, Eric S. Jackson

**Affiliations:** ^1^Institute of Neurobiology, National Autonomous University of Mexico, Querétaro, Mexico; ^2^Department of Psychology, New York University, New York, NY, United States; ^3^Music and Audio Research Lab, New York University, New York, NY, United States; ^4^Center for Music, Language and Emotion, New York University, New York, NY, United States; ^5^Department of Speech-Language Pathology, Duquesne University, Pittsburgh, PA, United States; ^6^Department of Communicative Sciences and Disorders, Michigan State University, East Lansing, MI, United States; ^7^Department of Communicative Sciences and Disorders, New York University, New York, NY, United States

**Keywords:** stuttering adult, interoception, speech synchronization, auditory-motor integration, remotely assessments

## Abstract

Stuttering is a neurodevelopmental speech disorder associated with motor timing that differs from non-stutterers. While neurodevelopmental disorders impacted by timing are associated with compromised auditory-motor integration and interoception, the interplay between those abilities and stuttering remains unexplored. Here, we studied the relationships between speech auditory-motor synchronization (a proxy for auditory-motor integration), interoceptive awareness, and self-reported stuttering severity using remotely delivered assessments. Results indicate that in general, stutterers and non-stutterers exhibit similar auditory-motor integration and interoceptive abilities. However, while speech auditory-motor synchrony (i.e., integration) and interoceptive awareness were not related, speech synchrony was inversely related to the speaker’s perception of stuttering severity as perceived by others, and interoceptive awareness was inversely related to self-reported stuttering impact. These findings support claims that stuttering is a heterogeneous, multi-faceted disorder such that uncorrelated auditory-motor integration and interoception measurements predicted different aspects of stuttering, suggesting two unrelated sources of timing differences associated with the disorder.

## Introduction

Stuttering, a neurodevelopmental speech disorder that impacts approximately 70 million individuals globally (Bloodstein et al., 2021), can have a significant negative impact on educational, occupational, and social achievement (Tichenor and Yaruss, 2019). Some accounts of stuttering associate the disorder with deficits in speech motor timing (Alm, 2004; Ludlow and Loucks, 2004; Etchell et al., 2014; Etchell et al., 2015). One line of research demonstrates that stutterers^1^ exhibit compromised auditory-motor integration, particularly regarding sensorimotor synchronization (SMS) abilities (Falk et al., 2015; van de Vorst and Gracco, 2017; Sares et al., 2019). Still, sensorimotor accounts cannot entirely explain this multifactorial disorder by themselves. Other work points to the involvement of interoception, or the awareness of internal body sensations, particularly with respect to how interoception relates to the anticipation of stuttering events (Garcia-Barrera and Davidow, 2015; Rodgers and Jackson, 2021). Importantly, interoception has been linked to timing skills (Craig, 2009; Meissner and Wittmann, 2011), and other disorders impacted by timing deficits, such as Tourette’s syndrome, are associated with compromised interoceptive systems (Mioni et al., 2016; Vicario and Felmingham, 2018; Graziola et al., 2020; Vicario et al., 2020). Thus, auditory-motor integration and interoception emerge as two cognitive features, both related to motor timing, and both potentially related to stuttering behavior. This invites the following question: do atypical auditory-motor integration and interoception derive from the same anomalous mechanism or do they reflect two independent sources of timing deficits? To this end, the current study examined the relationships between auditory-motor integration, interoceptive awareness, and self-reported stuttering severity. Remote assessment was implemented because of the benefits of using such methodologies in telepractice settings, especially for specific clinical populations.

### Auditory-Motor Integration and Stuttering

Both adult and child stutterers exhibit auditory-motor integration skills that differ from non-stuttering peers (Cai et al., 2012, 2014; Max and Daliri, 2019; Kim et al., 2020), indicating that they have difficulty using auditory feedback to guide the planning and execution of articulatory movements. Most prior studies used an auditory feedback perturbation paradigm in which formants are shifted in near-real time. In response, speakers shift their articulators to achieve formant patterns in the opposite direction to compensate for the auditory perturbation. During these tasks, the magnitude of stutterers’ responses is lower than that of non-stuttering speakers (Cai et al., 2012; Max and Daliri, 2019; Kim et al., 2020). Cai et al. (2014) examined responses to auditory feedback perturbations during connected speech. While the magnitude of responses differed only subtly between stutterers and non-stutterers, the stutterers still showed delayed responses, indicating a relative slowness in auditory-motor integration.

External timing, or the ability to synchronize to external cues, has been assessed using auditory-motor synchronization tasks in which participants are instructed to align rhythmic motor acts such as finger tapping with a cue such as a metronome (for review, see Repp and Su, 2013). Auditory-motor integration is then determined by measuring the variance, or asynchrony, between participants’ movements and the external cue. While various forms of external signals reduce stuttering (e.g., speaking in unison or with a metronome; for review, see Andrews et al., 1983), both adults and children who stutter exhibit differences in sensorimotor synchronization compared to non-stuttering controls (Falk et al., 2015; van de Vorst and Gracco, 2017; Sares et al., 2019; though see Hilger et al., 2016 for a lack of significant differences). In the only two studies that assessed sensorimotor synchronization in the speech domain, Boutsen et al. (2000) found that stutterers were more variable when producing syllables timed to a metronome, whereas Max and Yudman (2003) reported similar patterns between stutterers and non-stutterers when the participants repeated a syllable timed to a metronome. These contradictory results suggest that auditory motor synchrony deficits only affect a subgroup of stutterers, different timing mechanisms may underlie the control of different motor effectors (e.g., vocal tract articulators or fingers), or that limited sample sizes [e.g., in Max and Yudman (2003)] are responsible for the inconsistent results.

Neurally, stuttering is associated with structural and functional differences in basal ganglia, supplementary motor area, cerebellum, and premotor cortex; these areas are all related to the processing of external timing (Etchell et al., 2014, 2015; Falk et al., 2015). In addition, stutterers exhibit differences in brain structure and function in the left arcuate fasciculus (Cai et al., 2014; Cieslak et al., 2015; Chow and Chang, 2017), a dorsal language pathway that connects the posterior portion of the temporal lobe and inferior (pre-)frontal lobe, and which underlies sensorimotor integration (Catani et al., 2005; Repp and Su, 2013). Further, SMS is associated with the left arcuate fasciculus in non-stutterers (Blecher et al., 2016). Conversely, in stutterers, auditory-motor synchronization is associated with the left inferior cerebellar peduncle, which indicates that stutterers may rely more heavily on cerebellar tracts, perhaps as a compensatory mechanism (Jossinger et al., 2022).

Auditory-motor integration and the ability to synchronize to an external rhythm are also critical aspects of language processing and learning. Assaneo et al. (2019) demonstrated that the general population can be split into two distinct groups: those who synchronize their speech to external rhythms [high synchronizers (HS)] and those who do not [low synchronizers (LS)]. They used the Spontaneous Synchronization to Speech (SSS) test, a simple, quick, and effective tool to measure external timing that can be delivered remotely (Assaneo et al., 2019). The SSS test requires participants to repeat a single syllable while listening to strings of random syllables with a given tempo. They found that a speaker’s synchronization group, as established by the SSS test, was predictive of brain structure and function in regions associated with auditory-motor integration. In particular, the volume of the left arcuate fasciculus differentiated the HS and LS groups (with HS showing higher values than LS), highlighting the key role of this white matter pathway in external synchronization. In addition, HS magnetoencephalography recordings showed increased brain-to-stimulus synchronization in (pre-)frontal lobe (Broca’s area) during passive listening of rhythmic speech (Assaneo et al., 2019). HS individuals also displayed cognitive advantages compared to LS individuals, as they performed better at a phonological word learning task (Saffran et al., 1996). The relationships between SSS scores, language learning outcomes, and brain structure and function in regions related to auditory-motor integration suggest that the SSS test may be sensitive to and predictive of individual differences related to speech disorders. Given previously observed differences in auditory-motor integration and the structure and function of the arcuate fasciculus in stutterers, it is reasonable to hypothesize that the SSS task may also predict stuttering severity. One of the main goals of the current work is to test this hypothesis.

### Interoceptive Awareness and Stuttering

Interoception may also be relevant to speech timing mechanisms and stuttering severity. Interoception refers to one’s awareness of internal body sensations, such as cardiac markers (heartbeat) and discomfort. Garcia-Barrera and Davidow (2015) proposed that interoception plays an important role in learning to anticipate stuttering events. Anticipation refers to the awareness that an upcoming speech will be stuttered, should that speech be executed as planned (Jackson et al., 2015). Apart from a cognitive awareness or awareness during speech planning that the upcoming speech will be stuttered, bodily changes or interoceptive awareness (e.g., the sensation that articulators will get “stuck”) may also signal to the stutterer that they are about to overtly stutter, for example, through increased tensing in articulators or increased physiological responses (see Perkins, 1990; Tichenor and Yaruss, 2019 for discussion of the feeling of getting stuck). Importantly, interoceptive awareness is closely related to the perception of time (Craig, 2009; Meissner and Wittmann, 2011), and disorders affected by timing, including Tourette’s syndrome and anxiety disorders, are also associated with atypical interoceptive awareness (Mioni et al., 2016; Vicario and Felmingham, 2018; Graziola et al., 2020; Vicario et al., 2020). Tourette’s syndrome and stuttering share many similarities: both begin in early childhood, involve differences in basal ganglia structure, are associated with involuntary movements, and are positively and negatively affected by dopamine antagonists and agonists, respectively (Maguire et al., 2020). Ganos et al. (2015) found that tic severity, as reflected by premonitory urges, was negatively correlated with interoceptive awareness. In Ganos et al. (2015) study, interoceptive awareness was measured using a mental tracking task in which participants reported the number of their heartbeats, and their reports were compared to the “actual” number of heartbeats recorded. Premonitory urges are proactive markers that reflect an awareness of upcoming tics and thus are similar in nature to the anticipation of stuttering events. Although this hypothesis naturally arises from the existing evidence, the link between interoception and stuttering remains empirically untested.

Neurally, the anterior insular cortex underlies interoception (Critchley et al., 2004; Seth et al., 2012; Critchley and Harrison, 2013). The anterior insula also underlies speech production and perception timing (Oh et al., 2014) and auditory temporal processing (Ackermann et al., 2001). Zarate and Zatorre (2008) found increased activation in the insula for tasks that require monitoring of auditory feedback, and the insula plays an important role in the integration of auditory and somatosensory feedback in rats (Rodgers et al., 2008) and humans (Woolnough et al., 2019). Musicians, who demonstrate greater speech-to-speech synchrony than non-musicians (Assaneo et al., 2019), exhibit greater causal interaction output from the insula (Luo et al., 2012). Thus, the insula is a common neural substrate for diverse processes including interoception, speech monitoring, auditory temporal processing, and sensorimotor integration.

The purpose of the current study was to examine the relationships between auditory-motor integration (i.e., speech-to speech synchrony), interoception, and self-reports of stuttering severity in adult stutterers using measures that can be administered remotely. Participants completed the SSS test and the Multidimensional Assessment of Interoceptive Awareness - 2nd Version (MAIA-2; Mehling et al., 2018). They also responded to two questions that indirectly assessed stuttering severity and impact. This methodology allowed us: (i) to study the extent to which differences in auditory-motor integration are generalized across the stuttering population; and (ii) to empirically assess whether interoceptive awareness is predictive of stuttering severity. Moreover, by testing whether auditory-motor integration (*via* the SSS test) and interoception are related to stuttering severity and to one another, we were able to test two competing hypotheses: (1) differences in auditory-motor integration and interoception observed in stutterers (as compared to non-stutterers) arise due to differences in one underlying, shared brain mechanism or (2) auditory-motor integration and interoception rely on independent systems, and stuttering can emerge due to differences in either system.

## Methods

### Participants

Two cohorts completed the online version of the SSS-test: 91 self-reported, adult stutterers and 110 adult non-stutterers. Given the remote nature of the protocol, several subjects (35 stutterers and 33 non-stutterers) were excluded for various reasons: noisy environment, speaking aloud instead of whispering, or not wearing headphones. The final cohorts comprised 77 non-stutterers (34 females; mean age 37; age range 20–70 years) and 56 stutterers (10 females; mean age 34; age range 19–71 years). Three stutterers did not report sex or age. The final stutterer cohort also completed the MAIA-2 and responded to two severity questions (see below).

All data were collected online. For the non-stutterers, all tasks and questionnaires were delivered *via* Amazon Mechanical Turk, a crowdsourcing platform that facilitates the acquisition of large datasets from the general population. Stutterers were recruited *via* emails distributed to the New York University (NYU) stuttering research database and the Michigan State University Stuttering Surveys database.

Participants from both cohorts were native English speakers with self-reported normal hearing and no neurological deficits. They were paid for taking part in the study and provided informed consent. All protocols were approved by the Institutional Review Boards at New York University and Michigan State University.

### SSS-Test

Participants completed the online accelerated version of the SSS test as described in Assaneo et al. (2019). During this test participants, wearing headphones, were presented with a 1-min audio file comprising a rhythmic stream of syllables. They were instructed to pay attention to the audio while concurrently and continuously whispering the syllable “tah.” Participants’ vocalizations were recorded through their computer’s microphone. Next, we established each individual’s degree of speech-speech synchrony by computing the phase locking value (see below for a detailed explanation of this measure) between the envelope of the produced and perceived acoustic signals. The instructions given to the participants were adapted from the original Assaneo et al. (2019) procedures following Kern et al. (2021). In the original Assaneo et al. (2019) study, no explicit instruction was given for the participants to synchronize their whispered speech to the external stimulus; instead, participants were instructed to pay attention to the stimulus and to try to correctly recall the perceived syllables. Here, as in Kern et al. (2021), participants were explicitly instructed to synchronize to the external auditory signal and syllable recall was neither assessed, nor instructed. In this version of the task, the external syllabic rate started at 4.3 Hz and was increased in steps of 0.1 Hz until reaching 4.7 Hz, the total duration of the audio was 60 s. The stream of syllables was synthesized using the software MBROLA text-to-speech with the American male voice diphone database (US2) at 16 kHz (Dutoit et al., 1996). All phonemes were equal in pitch. Pitch raised until 200 Hz and fell symmetrically, with the maximum at the middle of the phoneme’s duration. The spontaneous nature of the synchrony relies on the fact that, although participants cannot detect the 0.1 Hz increments in the external syllabic rate (Assaneo et al., 2019), high synchronizers still automatically adjust their spoken pace to the subtly accelerating speech input. This version has been shown to replicate the original bimodal distribution—as well as show differences between the groups in auditory-motor-related cognitive features (Kern et al., 2021). Still, this result has not been replicated with a remote version of the test. For this reason, we decided to include a remote group of non-stutterers speakers in the current study.

As in Assaneo et al. (2019), we developed an online version of the SSS test using oTree, a Python-based platform for the development of online experiments (Chen et al., 2016). This online version included several steps to ensure task compliance. Specifically, a microphone test phase was included before the volume adjustment phase and several restrictions were placed to ensure that participants did not skip any steps (e.g., a participant could not continue to the next page until completing what was asked of them). The online SSS test was presented to Amazon Mechanical Turk (AMT) participants as an HTML webpage that ran in Google Chrome. Participants were first presented with a summary of the task, then with an informed consent page, and upon acceptance, with the main task instructions.

In addition, all participants completed the musical training subscale of the Gold-MSI questionnaire (Müllensiefen et al., 2014). Years of musical training is one of the items of the subscale and was the parameter explored in this study. Previous work shows that, in the general population, music experience differs between HS and LS, with the former displaying more years of musical training overall (Assaneo et al., 2019; Rimmele et al., 2022). In addition, musical training is related to the microstructural properties of white matter tracts connecting auditory to motor regions, including the arcuate fasciculus (Halwani et al., 2011), such that HS and LS exhibit differences in this white matter tract (Assaneo et al., 2019).

To estimate the individuals’ degree of synchrony we computed the phase locking value (PLV) between the envelopes of the perceived and the produced speech signals. The PLV was estimated in time windows of 5 s with an overlap of 2 s, according to the following formula:


$$PLV=\frac{1}{T}\sum_{t=1}^{T}e^{i\left(\theta_{1}\left(t\right)-\theta_{2}\left(t\right)\right)}$$

ss where *t* is the discretized time, *T* is the total number of time points in the window, and θ_1_ and θ_2_ are the phase of the perceived and produced speech envelopes, respectively, computed by applying the Hilbert transform on the corresponding signals. The PLVs were averaged across windows. This measurement is very robust to sudden discontinuities in the synchrony related, for example, to the subjects’ inhalation periods or to plausible blocks related to stuttering.

It is important to mention that the obtained individual’s synchronization measurement is robust to the attentional state of the participant. It has been shown that the outcome is related to brain structural features of the subject and that is stable across different sessions (Assaneo et al., 2019). Furthermore, the synchrony is spontaneous given that the subjects do not detect the increments in the external rate (neither the lows nor the high synchronizers) but they still automatically align their production rate (see Supplementary Figure 2 in Assaneo et al., 2019). So, as long as participants are correctly performing the test (i.e., continuously whispering at a rate above 2 Hz), the outcome will not reflect the level of commitment to the task.

### Multidimensional Assessment of Interoceptive Awareness—2nd Version (MAIA-2)

Participants from the stuttering cohort completed the MAIA-2 remotely *via* Qualtrics. The MAIA-2 is a 32-item self-report, state-trait questionnaire that measures multiple dimensions of interoception, including *Noticing, Not-Distracting, Not-Worrying, Attention Regulation, Emotional Awareness, Self-Regulation, Body Listening*, and *Trust*. Participants responded to each MAIA-2 item (e.g., “When I am tense I notice where the tension is located in my body,” “I can refocus my attention from thinking to sensing my body”) using a six-point Likert scale (0 = never, 5 = always). The MAIA is a valid and reliable self-report measure of interoceptive body awareness (Mehling et al., 2012), and the MAIA-2 has improved psychometric properties (Mehling et al., 2018). We used the total score, it is not norm-referenced, it can be calculated by summing or averaging the eight subscale scores. Several recent studies included the total MAIA score as an outcome variable (e.g., Donadeo et al., 2021; Gaggero et al., 2021; Smith et al., 2021). We compared our results with those from Mehling et al. (2018), which included 1,090 participants (18–69 years of age) who could comprehend English and were visitors to a residency project at the Science Museum in London, using an unpaired *t*-test.

### Severity Questions

Stuttering severity and impact were assessed using two Likert scale questions: (1) “How severe would other people rate your stuttering?” [severity as perceived by others (SPO)]; and (2) “Overall, how much does stuttering impact your life?” [stuttering impact (SI)]. These are common questions to ask in an evaluation or during therapy. Severity question 1 reflects the stutterers’ perceptions of how their listeners might perceive the severity of their stuttering based on observable behaviors. We view this rating as an indirect measure of overt severity (i.e., what the listener hears/sees). Although this measure is effectively filtered through the life experiences of the stutterer, in our clinical experience, adult stutterers are skilled at judging how much of their stuttering “comes to the surface.” Severity question 2 reflects the impact that stuttering has on the stutterer’s life. Participants responded by selecting one of five responses: mild, mild-moderate, moderate, moderate-severe, or severe. Other groups (O’Brian et al., 2004; Gunn et al., 2019) have used similar approaches in lieu of more deeply assessing severity.

### Statistical Analyses

All analyses were run in Matlab 2020b. Gaussian mixture distribution models with k components were adjusted to the synchronization measurement distribution, using the diagonal covariance matrix and allowing a maximum of 140 iterations (McLachlan and Peel, 2000). Normalized Akaike’s Information Criterion (AIC) values were calculated for each of the estimated models. The lowest AIC value was used to select among the set of possible models (i.e., *k* = 1 or *k* = 2) describing the data. We used Mann–Whitney–Wilcoxon test for between-subject comparisons and the Spearman correlation coefficient to assess the relationships between variables. Uncorrected two-sided *p*-values are reported and all tested comparisons and correlations are made explicit in the text.

## Results

**Figure 1:**
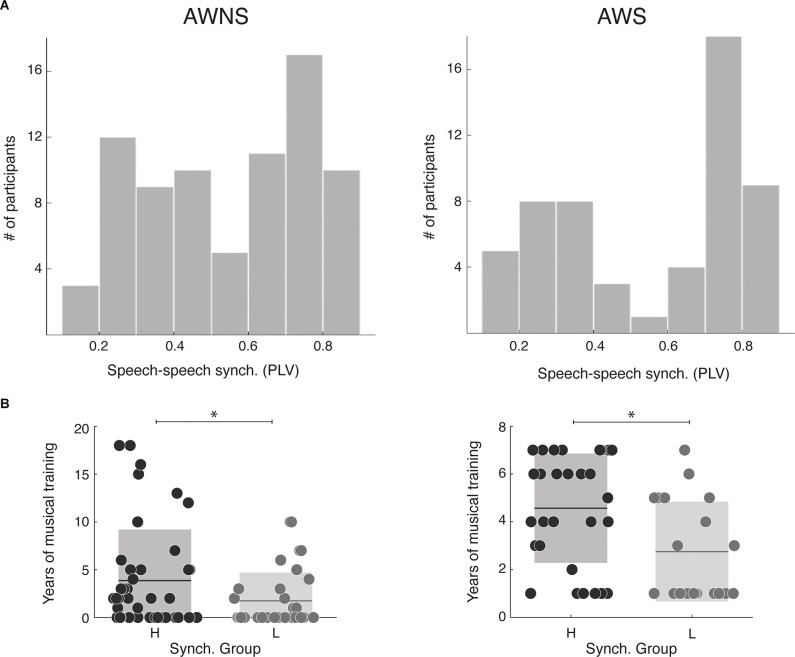
SSS-test outcome. **(A)** Distribution of the speech-to-speech synchronization measurements (left: 77 adult stutterers; right: 56 adult non-stutterers). For both cohorts, the model better adjusting the data is a Gaussian mixture distribution with two components (Stutterers: Component 1, mixing proportion: 0.57, mean: 0.78; Component 2, mixing proportion: 0.43, mean: 0.29. Non-stutterers: Component 1, mixing proportion: 0.54, mean: 0.73; Component 2, mixing proportion: 0.46, mean: 0.34). **(B)** Years of musical training for each synchrony group. On the leftnon-stutterers, on the right the stutterers. For both populations, high synchronizers have significantly more years of training than lows (Mann-Whitney-Wilcoxon test, non-stutterers: two-sided *p* = 0.046, stutterers: two-sided *p* = 0.0038). *p > 0.05.

First, we explored whether the distribution of the synchronization measurements obtained with the SSS-test significantly differs between cohorts. In both cohorts, participants continuously whispered the “tahs” with no silent gaps above 3 s and with a mean syllabic rate above 2 Hz, and a difference was not observed (*N*_stutterer_ = 56, *N*_non-stutterer_ = 77, Mann-Whitney-Wilcoxon test, two sided *p* = 0.8). Next, we investigated if the bimodal distribution previously reported in the general population for the SSS-test was shown in this sample of adult stutterers. We qualitatively compared the obtained results by computing the histograms of the synchronization measurements. As depicted in Figure 1A a bimodal distribution seems to be present in both cohorts. In order to quantify this observation, for each dataset, we applied two different Gaussian mixture distribution models (with 1 and 2 components) using the Akaike Information Criterion (AIC) to select the best fit. For both samples, the model with the lowest AIC was the one with two components (stutterers: AIC_1_ = 5.32 and AIC_2_ = −46.7; non-stutterers: AIC_1_ = −11.9 and AIC_2_ = −33.7), further confirming the bimodal nature of the distributions. Given these results, we divided the stutterers into two groups, high and low synchronizers (HS/LS) by applying a k mean clustering algorithm with two clusters to the obtained PLV measurements. Participants with a PLV above 0.55 were classified as HS, and participants with a PLV below 0.55 were classified as low synchronizers LS (stutterers: *N*_HS_ = 32 *N*_LS_ = 24; non-stutterers: *N*_HS_ = 41 *N*_LS_ = 31). Previous work shows that, in the general population, music experience differs between high and low synchronizers, with the former displaying more years of musical training overall (Assaneo et al., 2019). Here, we replicated this result and showed that it extends to adult stutterers (Mann-Whitney-Wilcoxon test, non-stutterers: two-sided *p* = 0.046, stutterers: two-sided *p* = 0.0038, see Figure 1B). Taken together, these results suggest that the brain mechanism underlying the bimodal distribution in stutterers is similar to that of the general population.

Second, we analyzed the interoceptive awareness results. First, we noticed that the distribution of this measurement was not bimodal (see Supplementary Figure 1) and as we did for the auditory-motor synchrony, we compared stutterers against the general population. For this purpose, we used the general population results reported in Mehling et al. (2018), and no difference was observed between stutterers and non-stutterers (unpaired, two-tailed *t*-test, *t* = 0.98, *p* = 0.33, Cohen’s *d* = 0 0.16). Next, we explored whether stutterers who were high synchronizers vs. low synchronizers reported different levels of interoceptive awareness. No significant difference was found between groups (Mann-Whitney-Wilcoxon test, two-sided *p* = 0.38).

**Figure 2:**
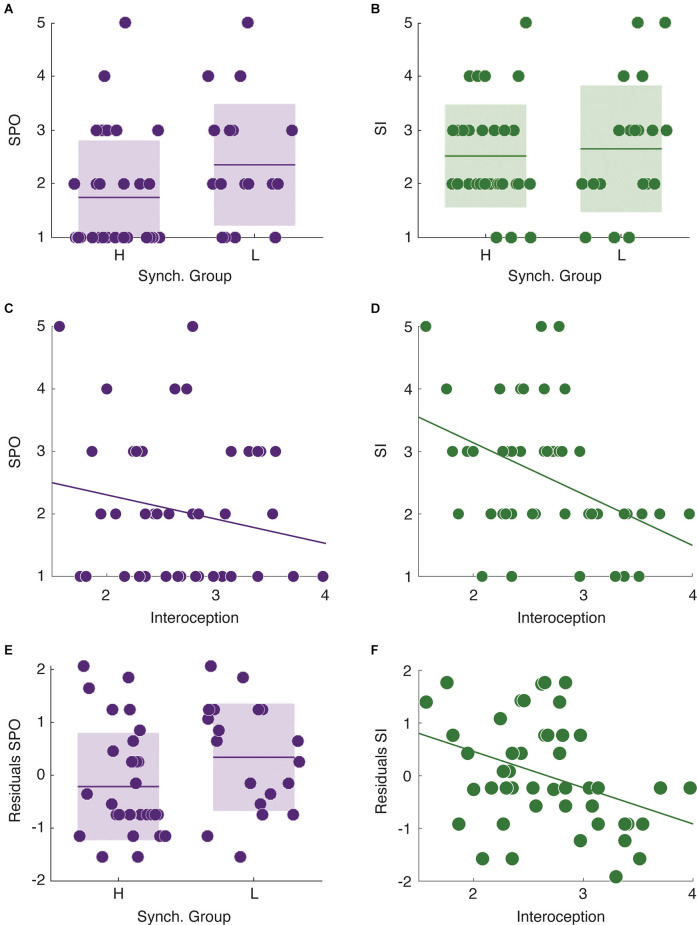
Relationships between individual cognitive features and the different levels of self-reported stuttering severity.** (A)** Low synchronizers show higher SPO values than HS. **(B)** No significant difference between synchrony groups in SI. **(C)** Interoception does not correlate with SPO. **(D)** There is a significant negative correlation between interoception and SI. **(E)** The residuals of the linear regression using SPO as dependent variable and SI as independent still differentiate between high and low synchronizers. **(F)** The residuals of the linear regression using SI as a dependent variable and SPO as the independent correlate with the interoception measurements. *p < 0.05.

Finally, we focused on the relationship between speech auditory-motor synchronization, interoceptive awareness, and self-reported stuttering severity in stutterers (for a complete description of the stuttering related self-report results see Supplementary Figure 2). More precisely, we studied whether synchrony group and level of interoceptive awareness predicted the two self-reported measures of observable severity and impact (SPO and SI, respectively). We found no significant difference in SI between high and low synchronizers (Mann-Whitney-Wilcoxon test, two-sided *p* = 0.73, Figure 2B). However, LS reported significantly higher SPO values than HS (Mann-Whitney-Wilcoxon test, two-sided *p* = 0.033, Figure 2A). This pattern was reversed for the level of interoception. While the interoception level significantly predicted SI (Spearman correlation, *r* = −0.42, *p* = 0.003, Figure 2D), it did not correlate with SPO (Spearman correlation, *r* = −0.17, *p* = 0.26, Figure 2C).

Although SI and SPO were selected as indicators of different aspects of self perceived stuttering severity, they could be conflated given that both measure stuttering experience albeit in different forms. Thus, to further evaluate the previously reported results, we assessed if the observed relationships remained significant while interchangeably regressing out the measurements from each other (SPO, SI) and examining the residuals. We found that the residuals after adjusting the linear regression with SPO as the dependent and SI as the independent variable still differentiated between high and low synchronizers (Mann-Whitney-Wilcoxon test, two-sided *p* = 0.043, Figure 2E). Similarly, after regressing out SPO from SI, the residuals still correlated with the interoception scores (Figure 2F, Pearson correlation, *r* = −0.36, *p* = 0.012).

Given that we performed comparisons between unbalanced groups in terms of gender (56 stutterers with 10 females), we explored for gender differences in our assessments. No significant differences between female and male stutterers were found in either (Mann-Whitney-Wilcoxon test, two-sided; Interoception: *p* = 0.08, PLV: *p* = 0.49, SPO: *p* = 0.35, SI: *p* = 0.9). Only interoception awareness showed a trend towards higher values for males than females (mean_FEM_ = 2.4, mean_MAL_ = 2.7).

## Discussion

In the current study, we explored the relationships between speech synchronization (used as an indirect measure of auditory-motor integration) and interoceptive awareness, as well as general measures of self-reported stuttering severity and impact. We found that speech synchronization and interoceptive awareness are uncorrelated and do not significantly differ between stutterers and non-stutterers, but they predict two different measures of the stuttering experience. Taken together, our pattern of results suggests that impairment in one of two unrelated timing mechanisms is enough to give rise to stuttering.

We tested whether the bimodal distribution seen in speech-to-speech synchronization abilities in the general population is also present in stutterers. Results indicated that, just as in the general population, there are two distinct groups reflecting differences in synchronization ability among stutterers: High Synchronizers (HS) and Low Synchronizers (LS). HS speakers synchronize their speech to an external rhythm, whereas LS speakers do not (Assaneo et al., 2019). Also consistent with findings from the general population (Rimmele et al., 2022), we found that differences in synchronization were related to musical training: HS individuals demonstrated more years of musical training than LS individuals. Interestingly, there were no differences between stutterers and non-stutterers on speech-specific SMS based on the SSS test, which is in line with Max and Yudman (2003). It may be that the lack of differences was due to the relatively low complexity of the SSS test and that more complex speech SMS tasks would reveal differences in SMS. It is also possible that there was a rhythm effect which mitigated group differences. It is unclear, however, why a rhythm effect would be specific to the speech domain, as several studies revealed non-speech SMS differences between stutterers and non-stutterers (Falk et al., 2015; van de Vorst and Gracco, 2017; Sares et al., 2019; though see Hilger et al., 2016 for a lack of significant differences). Future studies can tease apart details related to domain-specific and domain-general differences.

Participants in the LS group believed that listeners found their stuttering to be more severe compared to the reports of individuals in the HS group. This means that stutterers who demonstrated a decreased ability to synchronize to an external timing signal, that is, those who exhibit reduced auditory-motor speech integration abilities, perceived that their stuttering is more severe to their listeners. Thus, the current findings show a link between auditory-motor integration abilities and the ability of a stutterer to report how their stuttering is perceived by others. Future studies could examine the relationships between the SSS test and other measures of observable stuttering behavior, including other speaker-based measures (O’Brian et al., 2004; Gunn et al., 2019) and listener-based measures (Riley, 2009).

Together, these findings suggest that similar mechanisms underlie the synchronization group distinction in stutterers and non-stutterers. This suggests that in LS, stuttering may be related to differences in the microstructural properties in the arcuate fasciculus (Assaneo et al., 2019) or in the insula, which has been associated with the integration of auditory and somatosensory feedback in rats (Rodgers et al., 2008) and humans (Woolnough et al., 2019), as well as with increased monitoring of auditory feedback (Zarate and Zatorre, 2005). Findings further suggest that proposals about differences in auditory-motor integration should not be generalized across all stutterers. This is consistent with multifactorial accounts of stuttering postulating that different factors, or different combinations of factors, contribute to stuttering in different individuals (Adams, 1990; Walden et al., 2012; Smith and Weber, 2017). For instance, differences in auditory-motor integration may contribute to stuttering in some individuals, whereas in speakers with intact auditory-motor integration abilities, timing (e.g., Alm, 2004; Etchell et al., 2015) or inhibition (e.g., Markett et al., 2016; Neef et al., 2018) differences may contribute to stuttering.

Interoceptive awareness did not differ between stutterers and non-stutterer participants. However, stutterers with greater interoceptive abilities reported that stuttering had less impact on their lives compared to those individuals with lower interoceptive abilities. This indicates that stutterers who are more “in tune” with internal body sensations, such as heartbeat and physical discomfort, are less likely to report that stuttering significantly impacted their life. It may be that increased awareness of internal body sensations facilitates the processing of responses to stuttering, especially prior to overt speech. Interoception abilities likely play a role in the anticipation of stuttering events (Garcia-Barrera and Davidow, 2015; Rodgers and Jackson, 2021), so knowledge of upcoming speech difficulties (e.g., being more in tune with when one is going to stutter) may help a person respond productively when sensing that stuttering will occur. For example, they may be able to be more mindful so that they can implement more adaptive responses (Jackson et al., 2015). This could explain why interoceptive awareness correlates with stutterers’ ratings of the impact of stuttering on their lives but not with their rating of severity of stuttering as perceived by others.

Interoceptive awareness is associated with conditions involving impaired timing such as Tourette’s syndrome and anxiety disorder (Vicario and Felmingham, 2018; Martino et al., 2019; Vicario et al., 2020). Stuttering shares many characteristics with these disorders, including involuntary movements, dopamine imbalance, and heightened physiological responses. However, we did not find a relationship between interoceptive awareness and synchronization skills, which may be due to the differentiation between internal vs. external timing. The SSS test assessed external timing, or the ability to synchronize to an external rhythm. In contrast, interoceptive awareness may be more related to *internal* timing, or the ability to generate a rhythm without external facilitation, because it relates to the perception of internal states and experiences. This would be consistent with studies that show compromised structure and function in brain areas responsible for internal timing (e.g., basal ganglia, supplementary motor area) in both adults and children who stutter (Alm, 2007; Jiang et al., 2012; Chang and Zhu, 2013; Etchell et al., 2014, 2015). Future studies could examine the relationship between interoceptive awareness and internal timing to determine whether this distinction exists.

Our study suggests that while auditory-motor ability and interoceptive awareness are generally related to stuttering experience, they may be unrelated factors in stuttering. External timing or auditory-motor integration, as measured by the SSS test, is associated with severity as perceived by others whereas interoceptive awareness is associated with the stutterer’s perception of stuttering impact. This may reflect two distinct, non-overlapping mechanisms, which would be in line with the current thinking of stuttering as a heterogeneous, multi-faceted disorder (Alm, 2005; Smith and Weber, 2017). The same observable behavioral outcome (i.e., the stuttering event) could be caused by difficulty at two different stages of the neural processing that supports speech production.

In summary, we showed that: (i) stutterers, like non-stutterers, exhibit a bimodal distribution in terms of speech auditory-motor integration ability; (ii) stutterers do not differ from the general population in their interoceptive abilities; and (iii) each measurement correlates with a different self-report of the stuttering experience. These findings support the notion that stuttering is a heterogeneous, multi-faceted disorder such that impaired auditory-motor integration may contribute to stuttering in some individuals but not others. We also showed that two quick and effective assessments that can be delivered remotely potentially provide indicators of different types of severity—which may indicate non-overlapping mechanisms underlying stuttering. Future studies are needed to clarify the relationships between and among internal/external timing mechanisms, interoceptive awareness, and stuttering severity, as well as their underlying neural correlates.

## Data Availability Statement

All data needed to evaluate the conclusions in the article are present in the article and/or the Supplementary Information. Additional data related to this article may be requested from the authors.

## Ethics Statement

All protocols involving human participants were reviewed and approved by the Institutional Review Boards at New York University and Michigan State University. The patients/participants provided their written informed consent to participate in this study.

## Author Contributions

MA, EJ, and PR conceived and supervised the project and analyzed data. PR, EJ, ST, and JY conducted the experiments. MA and EJ wrote the manuscript. All authors contributed to the article and approved the submitted version.

## Conflict of Interest

The authors declare that the research was conducted in the absence of any commercial or financial relationships that could be construed as a potential conflict of interest.

## Publisher’s Note

All claims expressed in this article are solely those of the authors and do not necessarily represent those of their affiliated organizations, or those of the publisher, the editors and the reviewers. Any product that may be evaluated in this article, or claim that may be made by its manufacturer, is not guaranteed or endorsed by the publisher.

## Footnotes

^1^In this article, we have elected to use the terms “stutterers” and “non-stutterers.” This decision was done purposefully to align with identify-first language use. See Constantino (2018), for discussion.
