# Supplementary material for: The Relationship Between Auditory-Motor Integration, Interoceptive Awareness, and Self-Reported Stuttering Severity
Source: Front Integr Neurosci. 2022 May 6;16:869571. doi: 10.3389/fnint.2022.869571 (PMC9120354; doi:10.3389/fnint.2022.869571)
Supplement: Supplementary file 1 [file Data_Sheet_1.docx]

**Supplementary Materials**

**The relationship between auditory-motor integration, interoceptive awareness, and stuttering severity**

M. Florencia Assaneo, Pablo Ripollés, Seth E. Tichenor, J. Scott Yaruss and Eric S. Jackson

**Figure S1. Interception measurements distribution.** Distribution of the total scores calculated by averaging the eight subscale scores of the MAIA-2 obtained for the stutterer cohort (mean=2.67, std=0.53).

**Figure S2. Answers obtained with the two self-reports related to the stuttering experience.** On the right, results obtained for the question “How severe would other people rate your stuttering?”. On the left, results obtained for the question “Overall, how much does stuttering impact your life?”. Answers Likert scale: 1=mild, 2= mild-moderate moderate, 3=moderate-severe and 5=severe.
